# The Effect of* Poly(ADP-ribose) Polymerase-1* Gene 3′Untranslated Region Polymorphism in Colorectal Cancer Risk among Saudi Cohort

**DOI:** 10.1155/2016/8289293

**Published:** 2016-09-25

**Authors:** Abdullah M. Alhadheq, Jilani Purusottapatnam Shaik, Abdullah Alamri, Abdulrahman M. Aljebreen, Othman Alharbi, Majid A. Almadi, Faten Alhadeq, Nahla A. Azzam, Abdelhabib Semlali, Mohammad Alanazi, Mohammad D. Bazzi, Narasimha Reddy Parine

**Affiliations:** ^1^Genome Research Chair, Department of Biochemistry, College of Science, King Saud University, Riyadh, Saudi Arabia; ^2^College of Medicine, King Saud University, Riyadh, Saudi Arabia; ^3^Division of Gastroenterology, King Khalid University Hospital, King Saud University, Riyadh, Saudi Arabia; ^4^Division of Gastroenterology, The McGill University Health Center, Montreal General Hospital, McGill University, Montreal, QC, Canada; ^5^Department of Genetics, King Faisal Specialist Hospital and Research Center, Riyadh, Saudi Arabia

## Abstract

*Background*. DNA repair systems are essential for each cell to repair and maintain the genome integrity. Base excision repair pathway is one of the crucial pathways to maintain genome integrity and* PARP-1* plays a key role in BER pathway. The purpose of this study is to evaluate the association between polymorphisms in* PARP-1* 3′untranslated region (3′UTR) SNP rs8679 and its expression in colorectal cancer.* Methods*. Genotyping and gene expression were performed using TaqMan assays. The effects of age, gender, and tumor location were evaluated in cases and controls regarding the genotyping results. Resulting data was analyzed using SPSS software.* Results and Conclusions*. Genotyping analysis for SNP rs8679 showed decreased susceptibility to colorectal cancer at heterozygous TC allele and at minor allele C. Further this protective association was also observed in younger age patients (≤57), in female patients, and also in patients with tumors located at colon and rectum.* PARP-1* expression levels are significantly different in colorectal cancer compared to matched normal tissue. Our findings proved that the upregulation of* PARP-1* is associated with tumor progression and poor prognosis in Saudi patients with colorectal cancer, suggesting that* PARP-1* can be novel and valuable signatures for predicting the clinical outcome of patients with colorectal cancer.

## 1. Introduction

Colorectal cancer is one of the major neoplasms that affect the human digestive system. The incidence of CRC has been increased worldwide; it ranks 3rd among males and the second among females [1, 2]. In Saudi Arabia it ranks first among men and second in women. The combinations of genetics, epigenetics, and environmental factors make CRC as a multifactorial disease. Maybe due to westernized life style, the incidence and mortality of CRC have been increased in Saudi Arabia in past decade which resulted in highest mortality rates [3, 4].

DNA repair systems are essential for each cell to repair and maintain the genome integrity. Errors or abnormalities in the repair systems may lead to mutations which in turn cause the progression of cancer [5]. One of the key DNA repair pathways that play a role in cell viability is base excision repair pathway (BER) [6]. BER is a pathway which repairs error in DNA during cell division via activation of* PARP-1*, which initiates the BER pathway cascade.* PARP-1*, poly(ADP-ribose) polymerase, is 113 KDa nuclear protein [7].* PARP-1* plays a key role in repairing SS and DS breaks of DNA [8]. Furthermore,* PARP-1* expression is critical whether to influence or inhibit cancer progression [6].* PARP-1* overexpression may show both positive and negative effect cancer patients. In precancerous stage* PARP-1* might act as shield to carcinogenesis [9]. In contrast to this when a tumor has highly expressing* PARP-1*, it might protect the tumor from the DNA damaging treatment effects. In such cases* PARP-1* inhibitors play a key role in anticancer therapy [9, 10]. Several studies confirmed any variations or in absence of* PARP-1* gene can lead to errors in DNA repair, genetic instability, and modulation of gene transcription; thus it can enhance tumor development [11]. Previous studies reported that defects in* PARP-1* are enhancing cancer risk [12]. The* PARP-1* gene polymorphisms have been previously reported to be associated with risk in various carcinomas, including colon [13], lung cancer [14], bladder [15], prostate [16], head and neck [17], and breast cancer [18, 19]. SNP sites in 3′UTR could affect gene expression by blocking miRNA binding the target sites.* PARP-1* polymorphism rs8679 which is in 3′UTR region has previously studied in several cancers but there are conflicting reports about its role in cancer progression. In the present study we are intended to investigate the contribution of* PARP-1* 3′UTR SNP rs8679 (T3823C) genetic polymorphism and expression level in colorectal cancer among Saudi population.

## 2. Materials and Methods

### 2.1. Study Population

In this case-control study, a total number of 373 blood samples have been used. This includes 183 colorectal cancer and 190 matched normal samples. Sixty fresh colorectal cancer tissues and adjacent normal tissues from the same patient were collected in RNAlater solution for gene expression analysis (see Supplementary Table 1 in Supplementary Material available online at http://dx.doi.org/10.1155/2016/8289293). Samples and clinical data (age, gender, family history, smoking habits, tumor stage, grade, and location) were collected from King Khalid University Hospital (KKUH) in Riyadh, Saudi Arabia. IRB was obtained from Ethics Committee of College of Medicine King Khalid University Hospital, King Saud University. Five mL of blood obtained from patients and normal persons in an EDTA solution-containing tube and stored at −80°C until further use.

### 2.2. DNA Extraction and Genotyping

DNA was extracted from 200 *µ*L blood samples by using QIAmp DNA Blood Mini Kit (Qiagen, USA). The yield DNA concentration and purity were measured by the NanoDrop8000 spectrophotometer (Thermo Scientific).* PARP-1* SNP rs8679 was genotyped using TaqMan assay (Applied Biosystems) as previously described by [20].

### 2.3. Gene Expression

Colorectal cancer and adjacent normal tissues were collected from patients for RNA isolation. RNA was extraction from 20 mg of fresh tissue using Qiagen RNeasy Mini Kit (Qiagen, USA). cDNA was synthesized using the High Capacity cDNA Reverse Transcription Kit (Applied Biosystems, Foster City, CA). The resulting cDNA was used for estimation of the relative mRNA expression rate of* PARP-1* and* GAPDH* genes. TaqMan Gene Expression Assays (Applied Biosystems, Foster City, CA) were used for gene expression analysis of* PARP-1* and GAPDH and amplification was performed using an ABI 7500 fast real-time PCR system (Life Technologies, USA). Three replicates were used for each sample. The relative* PARP-1* expression levels were normalized to the GAPDH expression value. Data was evaluated using the comparative CT (2^−ΔCT^) method.

### 2.4. Statistical Analysis

The required sample size was calculated using Power and Sample Size Calculation Software Package (Vanderbilt University, Nashville, TN). Genotype and allelic frequencies of cases and control groups were compared using Pearson's goodness-of-fit chi-square. SPSS statistical software version 22 (SPSS, Chicago, IL, USA) was used to calculate chi-square values, odds ratios (OR), 95% confidence intervals (CI), and *p* values. Allele and genotype frequencies of* PARP-1* SNP rs8679 for Saudi populations were compared with HapMap populations. Allele frequencies of Saudi population and other populations were compared using pairwise chi-square (*χ* ^2^) tests as described by Alanazi et al. [21]. Survival curves were plotted by the Kaplan-Meier method and compared by the log-rank test. The survival data were evaluated by univariate Cox regression analyses. *p* < 0.05 was considered statistically significant. Mann–Whitney *U* test was performed to analyze the association between gene expressions.

## 3. Results

In the present study, we have evaluated the association 3′UTR region SNP of* PARP-1* gene (rs8679) with colorectal cancer in 183 cases and 190 age and gender matched controls of Saudi population. The clinical data was shown in Table 1. The genotype and allele frequencies distribution along with odds ratios and significance of rs8679 are shown in Table 2.

SNP rs8679 of* PARP-1* gene showed a statistically significant protective association with Saudi colorectal cancer patients. The genotype distributions of the analyzed SNP are as follows: 0.61 TT and 0.33 TC and 0.06 CC in cases but 0.47 TT and 0.45 TC and 0.07 CC in controls. The “TC” heterozygous allele posed about 0.56-fold lower risk with cases compared to the homozygous allele “TT” (OR: 0.56, CI: 0.368–0.871, *χ* ^2^ = 6.75; *p* < 0.009) (Table 2). The combination of “TC + CC” variants alleles genotypes also showed about 0.58-fold lower risk in cases, compared to the wild-type alleles (OR: 0.58; *χ* ^2^ = 6.62; CI: 0.387–0.881; *p* = 0.01007). Moreover, we also found significant protective association at minor allele C with colorectal cancer cases. The minor allele frequency is less in cases (23%) compared to controls (30%) and there was 0.695-fold lower risk in cases, compared to controls (*χ* ^2^ = 4.75; CI: 0.501–0.965; *p* = 0.02927). The CC genotype alone did not show any effect. The reasons for these differences remain unclear but may relate to the distribution rate of the alleles in the Saudi population.

### 3.1. Effect of Age and Gender on the Association of* PARP-1* SNP and CRC

To evaluate the association of* PARP-1* SNP rs8679 with age at cancer diagnosis and gender. Patients were classified based on median age of cancer diagnosis as ≤57 (*n* = 87) and >57 (*n* = 96) and genotype frequencies were compared with age matched healthy controls. The genotypic frequencies of both clusters are shown in Table 3.

Similar to overall study younger age patients showed more protective association at heterozygous TC genotype (OR: 0.466, CI: 0.245–0.886, *χ* ^2^ = 5.51; *p* < 0.0189) and TC + CC combined genotype (OR: 0.527, CI: 0.288–0.964, *χ* ^2^ = 4.36; *p* < 0.03682) showed protective effect compared to TT homozygous genotype (Table 3). However, in older aged patient's genotype and alleles did not show any associations with colorectal cancer risk (Table 3). After Bonferroni correction only TC genotype showed protective association against colorectal cancer in below 57-year-old age patients (Table 3).

Interestingly this SNP rs8679 showed statistically significant protective association only with the female gender and has no association with the male gender (Table 4). The rs8679 SNP showed statistically significant protective association against colorectal cancer risk with all alleles in females; the genotype distributions for TT, TC, and CC were 0.42, 0.49, and 0.09 in controls but 0.67, 0.30, and 0.03 in cases (Table 4). The heterozygous “TC” genotype frequency is less compared to homozygous allele “TT,” which posed 0.39-fold protective effect in colorectal cancer cases (*χ* ^2^: 7.32; CI: 0.193–0.775; *p* = 0.00682). In addition, the homozygous variant “CC” genotype frequency was also significantly low in female patients (0.03) compared to controls (0.09) and CC genotype showed significant protective effect in colorectal cancer female cases (OR: 0.181, *χ* ^2^: 5.04; CI: 0.035–0.927; *p* = 0.02482). The combination of heterozygous and variant genotypes TC + CC exhibited 0.353-fold lower risk in female cases, compared to female controls (*χ* ^2^: 9.43; CI; 0.180–0.692; *p* = 0.00213). Along with this the minor allele “C” frequency is significantly low in female colorectal cancer patients (0.18) compared to gender matched controls (0.34) (OR: 0.425, *χ* ^2^ = 9.79; CI: 0.247–0.732; *p* = 0.00176). Even after Bonferroni correction we found significant protective association in female colorectal cancer patients with TC, CC, and TC + CC genotypes and also at C allele (Table 4).

### 3.2. Association of* PARP-1* SNP rs8679 with CRC Risk Based on Tumor Location

To conduct the association of SNP rs8679 with tumor location of colorectal cancer we have stratified samples as colon and rectum based on tumor location. Interestingly SNP rs8679 SNP showed statistically significant protective association in both colon and rectum in CRC patients.

Patients with tumor located in colon area showed significantly lower risk (0.57-fold) with heterozygous genotype “TC” when compared with healthy individuals (*χ* ^2^: 4.69; CI: 0.345–0.950; *p* = 0.03028). The minor allele C frequency is also significantly low in patients with tumor located in colon area when compared to healthy controls (*χ* ^2^: 3.96; CI: 0.455–0.995; *p* = 0.04668) (Table 5). Patients with tumor located in rectum area showed significant protective effect at heterozygous TC genotype (*χ* ^2^: 4.74; CI: 0.277–0.940; *p* = 0.02954) and TC + CC (*χ* ^2^: 4.78; CI: 0.295–0.940; *p* = 0.02873) when compared with control samples (Table 5). But after Bonferroni corrections none of the genotypes showed significant association with colorectal cancer risk in patients with tumor located in colon and rectum areas (Table 5).

### 3.3. Genotype and Allele Frequencies of* PARP-1* SNP rs8679 Variant in Saudis and Other Populations

We compared the genotypic and allelic frequencies of the* PARP-1* SNP rs8679 in a normal healthy Saudi population with those of subjects in the HapMap project study groups. The allelic frequencies of rs8679 were significantly different with most of the populations except CEU (*p* = 0.052439), GIH (*p* = 0.344746), and TSI (*p* = 0.401752) populations. Tuscans in Italy (TSI, *χ* ^2^ = 0.703075) population showed more similarity with Saudi population in allelic frequencies (Table 6).

### 3.4. *PARP-1* Gene Expression

**Figure 1:**
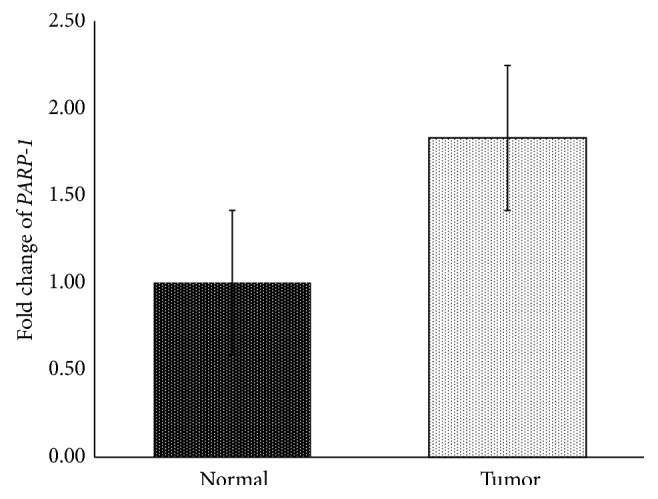
Relative* PARP-1* mRNA expression in colorectal cancer and adjacent normal samples (*n* = 56).

The present study has also investigated* PARP-1* expression levels in colorectal cancer tissues versus those of matched healthy tissues of the same patient. The results are shown in Figure 1. On the average,* PARP-1* mRNA expression level (mean = 1.82 ± 0.30) was significantly higher in tumor tissues than in healthy tissues. Mann–Whitney *U* test results showed significant difference in expression levels of tumor versus normal tissues (Mann–Whitney *U* = 913, *p* = 0.02). Based on median expression level (1.03), colorectal cancer patients were divided into two groups. The first group comprised 24 cases which showed low-expression and the remaining 32 cases were included into high-expression group.

### 3.5. Impact of* PARP-1* Expression on Prognosis of Colorectal Cancer

**Figure 2:**
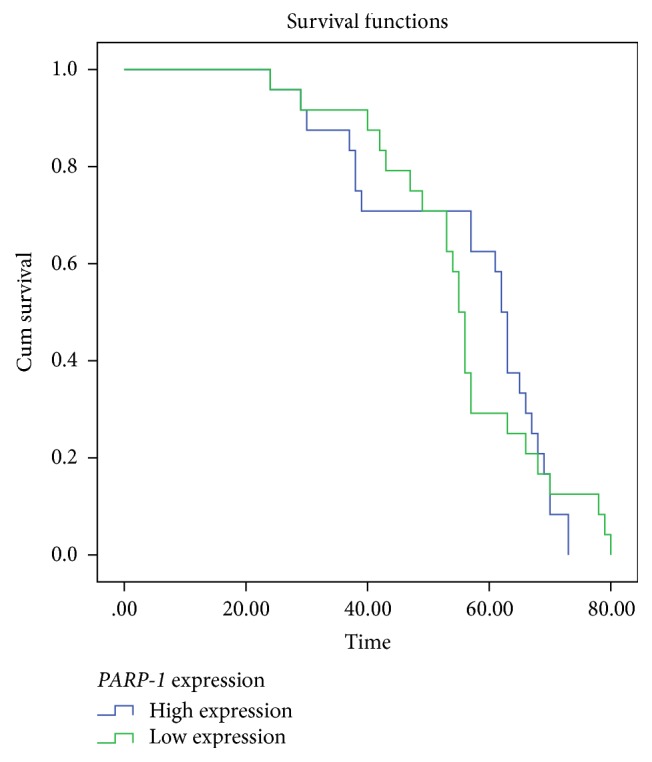
Kaplan-Meier analysis of DFS according to* PARP-1* expression level of colorectal cancer patients (*p* < 0.012) in Saudi cohort (time in years).

Kaplan-Meier method and log-rank test were used to evaluate the differences of overall survival between low-expression group and high-expression group. Kaplan-Meier analysis revealed that* PARP-1* expression levels were not associated with survival rate in Saudi colorectal cancer patients (55 versus 62, *p* < 0.873, Figure 2).

We also performed Cox regression to determine which clinical and demographic parameters were significantly associated with* PARP-1* expression level. Univariate analyses were used to assess whether the* PARP-1* expression level and various clinicopathological conditions were independent prognostic parameters of colorectal cancer patient outcomes. The results of analysis are shown in Table 7. A univariate analysis of the prognosis factors with a Cox proportional hazards model confirmed that low has-miR-145 (HR = 3.083, 95% CI: 1.944–8.24, *p* = 0.026) expression levels were significantly independent predictors of poor survival in colorectal cancer.

## 4. Discussion

*PARP-1* is very highly expressed enzyme and it is the most identified and the well-characterized* PARP* of all the 17-member PARP family. In PARP family* PARP-1* is accountable for majority of PARP activity, along with* PARP-2* [22].* PARP-1* detects and binds, with high affinity, to DNA strand breaks and then it interacts and activates several proteins required for DNA damage repair and recruits these proteins at the site of breakage [23]. Beside DNA repair function,* PARP-1* is involved in many cellular processes including conserving genomic stability, DNA synthesis, cell cycle regulation, telomere homeostasis, inflammation, and malignancy [24]. One of the key variants is* PARP-1* rs8679 which is 3′UTR region and reported to have binding site for has-miR-145 and it is reported to be associated with increased risk of breast cancer [25].

In the present study we observed a significant protective association of* PARP-1* rs8679 genotypes in colorectal cancer patients. Our genotyping results are contradicting with previous studies conducted in breast cancer [25, 26] and hepatocellular carcinoma [27]. Teo et al. [25] reported that rs8679 was significantly associated with increased breast cancer risk in individuals homozygous for the variant. Along with this they have reported that* PARP-1* rs8679 SNP has binding site for has-miR-145 and also been predicted to have increased ΔΔG, whereas variant allele ΔG is less negative than the wild-type allele ΔG. This may reduce chances of has-miR-145 binding to* PARP-1* mRNA 3′UTR which may enhance* PARP-1* expression [25]. Zhai et al. [26] also reported that* PARP-1* promoter SNP rs8679 does not show any association with breast cancer patients in Chinese population. Guillot et al. [27] reported that significant association was not observed between the* PARP-1* rs8679 genotypes and its expression and activity. They reported that 2 cell lines (HepG2 and HepG2 2.2.15) have variant allele C of SNP rs8679; this is one of the possibilities in reduction to detect any differences in activity or expression based on specific genotype [27].

Stratification of samples based on clinical and demographic characteristics showed that rs8679 is showing reduced risk in colorectal cancer patients below 57-year-old patients (Table 3), in female patients (Table 4), in patients with tumor located in colon area (Table 5), and in rectum area (Table 5).

Our results provide the first evidence that the* PARP-1* rs8679 polymorphism was associated with a decreased risk of colorectal cancer in a Saudi population.

**Figure 3:**
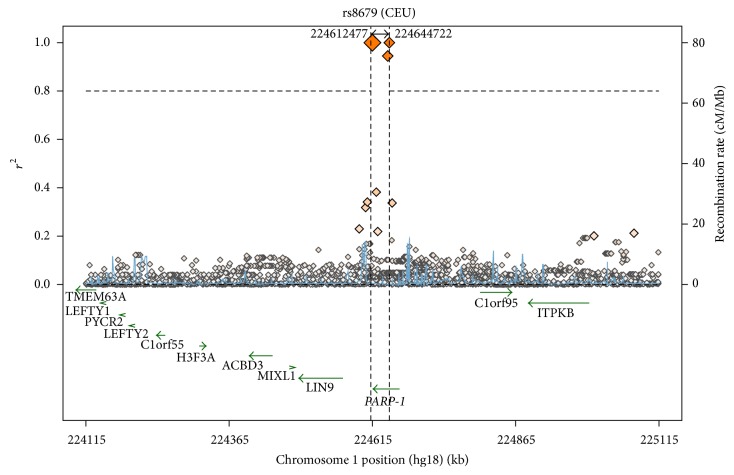
Regional linkage disequilibrium plot for the single nuclear polymorphism rs8679.

Comparison of rs8679 genotypic frequencies with other HAPMAP populations showed close association of Saudi population with Italian population (TSI) and Indian and central European populations (Table 6). We also generated a regional linkage disequilibrium (LD) plot using SNAP (SNP Annotation and Proxy Search, http://www.broadinstitute.org/mpg/snap/ldplot.php) for* PARP-1* rs8679. The LD plot indicated that there are multiple loci near rs8679 with high LD (*r* ^2^ > 0.8), which suggests that fine mapping is necessary to evaluate the genetic effect of* PARP-1* on cancer as well as functional studies (Figure 3). rs8689 is located at 5.3 kb upstream to 3′UTR region of* PARP-1* and is in LD (*r* ^2^ > 0.8) with* PARP-1* SNPs rs2271347 (*r* ^2^ = 1), rs61835377 (*r* ^2^ = 1), rs1805403 (*r* ^2^ = 1), and rs2793383 (*r* ^2^ = 0.945), which has been shown to be associated with increased risk for several cancers and other diseases.

In the present study, relative* PARP-1* expression is statistically significantly high in colorectal cancer tissue when compared to normal tissue (Figure 1). Several factors affecting* PARP-1* upregulation include* PARP-1* 3′UTR polymorphisms, transcription factors, and noncoding RNAs [19]. Our findings regarding* PARP-1* expression in colorectal cancer are consistent with previously published research in which* PARP-1* overexpression was also found in other types of cancer, including breast cancer [28], colorectal cancer [29, 30], prostate cancer [31], and glioblastoma [32].* PARP-1* expression was elevated throughout the patient-derived CRC samples.* PARP-1* is an attractive target for tumor detection because it is increased expression in a large number of cancers.* PARP-1* overexpression is believed to be due to the increased DNA damage occurring in genetically unstable cancer cells, rather than the activation of specific oncogenic pathways [33]. Nosho et al. [29] reported that* PARP-1* overexpression was correlated significantly with overexpression of *β-catenin*,* c-myc*,* cyclin D1*, and* MMP-7* in colorectal cancer.

In contrast to this Bai and Cantó [22] reported that rs8679 does not have any correlation with* PARP-1* expression; this is opposing our results, but they observed significantly higher expression of* PARP-1* gene in breast cancer.

*PARP-1* high-expression level was significantly associated with age, gender, and tumor stage (Table 7). The higher levels of* PARP-1* may be associated with poorer outcome. Our observations of increased expression of* PARP-1* in poor prognosis tumors do lend support to the view that PARP inhibitors might play a role in therapy for colorectal cancer patients.

In conclusion, our study assessed colorectal cancer predisposition with* PARP-1* gene 3′UTR SNP rs8679 in Saudi population.* PARP-1* rs8679 SNP showed significant protective effect with CRC risk and it did not correlate with susceptibility to colorectal cancer in Saudi population. Our findings support that the upregulation of* PARP-1* is associated with tumor progression and poor prognosis in Saudi patients with colorectal cancer, suggesting that* PARP-1* can be novel and valuable signatures for predicting the clinical outcome of patients with colorectal cancer.

## Supplementary Material

The details of samples used were provided in Supplementary Table 1.

## Figures and Tables

**Table 1:** Clinical characteristics of CRC cases.

Clinical characteristics	*N*
*Median age, years*	58
Range	21–78
*Gender*	
Male	110
Female	73
*Age*	
≤57	87
>57	96
*Tumor location*	
Colon	106
Rectum	65
*Stage at presentation*	
I	11
II	53
III	81
IV	38
Family history of cancer	32

**Table 2:** Genotype frequencies of *PARP-1* gene SNP rs8679 polymorphism in colorectal cancer and controls.

Genotype	Colon	Controls	OR	(95% CI)	*χ* ^2^ value	*p* value
TT	111 (0.61)	90 (0.47)	Ref			
TC	60 (0.33)	86 (0.45)	0.566	0.368–0.871	6.75	0.00936
CC	12 (0.06)	14 (0.07)	0.695	0.306–1.577	0.76	0.38242
TC + CC	72 (0.49)	100 (0.52)	0.584	0.387–0.881	6.62	0.01007
T	282 (0.77)	266 (0.70)	Ref			
C	84 (0.23)	114 (0.30)	0.695	0.501–0.965	4.75	0.02927

**Table 3:** Genotype frequencies of *PARP-1* gene polymorphism in colorectal cancer and controls based on age.

Genotype	CRC	Controls	OR	(95% CI)	*χ* ^2^ value	*p* value	Corrected *p* value^*∗*^
	Below 57						

TT	59 (0.68)	50 (0.53)	Ref				
TC	22 (0.25)	40 (0.42)	0.466	0.245–0.886	5.51	0.01890^*∗*^	**0.0378**
CC	6 (0.07)	5 (0.05)	1.017	0.293–3.532	0.00	0.97889	1
TC + CC	28 (0.32)	45 (0.47)	0.527	0.288–0.964	4.36	0.03682^*∗*^	0.07364
T	140 (0.80)	140 (0.74)	Ref				
C	34 (0.20)	50 (0.26)	0.680	0.415–1.115	2.35	0.12537	0.25074

	Above 57						

TT	52 (0.54)	40 (0.42)	Ref				
TC	38 (0.40)	46 (0.48)	0.635	0.350–1.153	2.24	0.13471	0.26942
CC	6 (0.06)	9 (0.10)	0.513	0.169–1.560	1.42	0.23369	0.46738
TC + CC	44 (0.46)	55 (0.58)	0.615	0.347–1.090	2.78	0.09531	0.19062
T	142 (0.74)	126 (0.66)	Ref				
C	50 (0.26)	64 (0.34)	0.693	0.446–1.077	2.66	0.10263	0.20526

^*∗*^Bonferroni corrected *p* value.

**Table 4:** Genotype frequencies of *PARP-1* gene polymorphism in colorectal cancer and controls based on gender.

Genotype	CRC	Controls	OR	(95% CI)	*χ* ^2^ value	*p* value	Corrected *p* value^*∗*^
	Male						

TT	62 (0.56)	59 (0.52)	Ref				
TC	38 (0.35)	48 (0.42)	0.753	0.432–1.313	1.00	0.31692	0.63384
CC	10 (0.09)	7 (0.06)	1.359	0.486–3.806	0.34	0.55777	1
TC + CC	48 (0.44)	55 (0.48)	0.830	0.491–1.406	0.48	0.48895	0.9779
T	162 (0.74)	166 (0.73)	Ref				
C	58 (0.26)	62 (0.27)	0.959	0.631–1.457	0.04	0.84292	1

	Female						

TT	49 (0.67)	31 (0.42)	Ref				
TC	22 (0.30)	36 (0.49)	0.387	0.193–0.775	7.32	0.00682^*∗*^	**0.01364**
CC	2 (0.03)	7 (0.09)	0.181	0.035–0.927	5.04	0.02482^*∗*^	**0.04964**
TC + CC	24 (0.33)	43 (0.58)	0.353	0.180–0.692	9.43	0.00213	**0.00426**
T	120 (0.82)	98 (0.66)	Ref				
C	26 (0.18)	50 (0.34)	0.425	0.247–0.732	9.79	0.00176^*∗*^	**0.00352**

^*∗*^Bonferroni corrected *p* value.

**Table 5:** Genotype frequencies of *PARP-1* gene polymorphism in colorectal cancer and controls based on tumor location.

Genotype	Tumor	Controls	OR	(95% CI)	*χ* ^2^ value	*p* value	Corrected *p* value^*∗*^
	Colon						

TT	64 (0.61)	90 (0.47)	Ref				
TC	35 (0.33)	86 (0.45)	0.572	0.345–0.950	4.69	0.03028^*∗*^	0.06056
CC	6 (0.06)	14 (0.7)	0.603	0.220–1.652	0.98	0.32134	0.64268
TC + CC	41 (0.39)	100 (0.52)	0.577	0.355–0.936	5.00	0.02533^*∗*^	0.05066
T	163 (0.78)	266 (0.70)	Ref				
C	47 (0.22)	114 (0.30)	0.673	0.455–0.995	3.96	0.04668^*∗*^	0.09336

	Rectum	Controls					

TT	41 (0.63)	90 (0.47)	Ref				
TC	20 (0.31)	86 (0.45)	0.510	0.277–0.940	4.74	0.02954^*∗*^	0.05908
CC	4 (0.06)	14 (0.7)	0.627	0.194–2.023	0.62	0.43167	0.86334
TC + CC	24 (0.37)	100 (0.52)	0.527	0.295–0.940	4.78	0.02873^*∗*^	0.05746
T	102 (0.78)	266 (0.70)	Ref				
C	28 (0.22)	114 (0.30)	0.641	0.399–1.027	3.45	0.06318	0.12636

^*∗*^Bonferroni corrected *p* value.

**Table 6:** Allele frequencies of *PARP-1* SNP rs8679 in Saudi and other populations.

Population	Number of samples	Freq. of “T”	Freq. of “C”	*χ* ^2^ value	*p* value
CEU	226	0.22 (49)	0.78 (177)	3.761721	0.052439
JPT	172	0.08 (13)	0.92 (159)	29.14925	<0.00001
YRI	226	0.02 (4)	0.98 (222)	66.0301	<0.00001
ASW	98	0.07 (7)	0.93 (91)	19.54286	<0.00001
CHB	82	0.05 (4)	0.95 (78)	20.77929	<0.00001
CHD	170	0.06 (11)	0.94 (159)	32.42167	<0.00001
GIH	176	0.26 (45)	0.74 (131)	0.892703	0.344746
LWK	180	0.01 (1)	0.99 (179)	60.63121	<0.00001
MEX	100	0.15 (15)	0.85 (85)	7.898509	0.004948
MKK	286	0.04 (12)	0.96 (274)	61.30637	<0.00001
TSI	176	0.34 (60)	0.66 (116)	0.703075	0.401752
SAUDI	190	0.3 (57)	0.7 (133)	Ref	

CEU = Utah residents with northern and western European ancestry from the CEPH collection; JPT = Japanese in Tokyo, Japan; YRI = Yoruba in Ibadan, Nigeria; ASW = African ancestry in southwest USA; CHB = Han Chinese in Beijing, China = CHD; GIH = Gujarati Indians in Houston, Texas; LWK = Luhya in Webuye, Kenya; MEX = Mexican ancestry in Los Angeles, California; MKK = Maasai in Kinyawa, Kenya; TSI = Tuscans in Italy; SAUDI = Saudi population residing in Riyadh region.

**Table 7:** Univariate analyses of *PARP-1* and different prognostic parameters on colorectal cancer.

Variables	Univariate analyses		
	HR	95% CI	*p* value
Age	2.41	0.89–8.54	0.033
Gender	3.54	1.24–10.09	0.018
Tumor location	2.15	0.79–5.85	0.134
TNM stage	3.233	1.14–9.19	0.028
*PARP-1* expression level	3.083	1.944–8.24	0.026
