# Supplementary material for: The Effect of Poly(ADP-ribose) Polymerase-1 Gene 3′Untranslated Region Polymorphism in Colorectal Cancer Risk among Saudi Cohort
Source: Dis Markers. 2016 Sep 25;2016:8289293. doi: 10.1155/2016/8289293 (PMC5055945; doi:10.1155/2016/8289293)
Supplement: Supplementary file 1 — The details of samples used were provided in Supplementary Table 1. [file 8289293.f1.docx]

**Supplementary Table 1:** Clinical characteristics of CRC cases used for gene expression study

| **Clinical characteristics** | **N** |
| --- | --- |
| **Gender** |  |
| Male | 34 |
| Female | 26 |
| **Age** |  |
| ≤57 | 28 |
| >57 | 32 |
| **Tumor location** |  |
| Colon | 39 |
| Rectum | 21 |
| **Stage at presentation** |  |
| I | 9 |

| II | 20 |
| --- | --- |
| III | 20 |
| IV | 11 |
